# Chemical Chaperone of Endoplasmic Reticulum Stress Inhibits Epithelial-Mesenchymal Transition Induced by TGF-*β*1 in Airway Epithelium via the c-Src Pathway

**DOI:** 10.1155/2017/8123281

**Published:** 2017-07-19

**Authors:** Heung-Man Lee, Ju-Hyung Kang, Jae-Min Shin, Seoung-Ae Lee, Il-Ho Park

**Affiliations:** ^1^Department of Otorhinolaryngology-Head and Neck Surgery, Korea University College of Medicine, Seoul, Republic of Korea; ^2^Department of Biomedical Sciences, Korea University Graduate School, Seoul, Republic of Korea; ^3^Medical Devices Clinical Trial Center, Guro Hospital, Korea University, Seoul, Republic of Korea

## Abstract

Epithelial-mesenchymal transition (EMT) is a biological process that allows epithelial cells to assume a mesenchymal cell phenotype. EMT is considered as a therapeutic target for several persistent inflammatory airway diseases related to tissue remodeling. Herein, we investigated the role of endoplasmic reticulum (ER) stress and c-Src in TGF-*β*1-induced EMT. A549 cells, primary nasal epithelial cells (PNECs), and inferior nasal turbinate organ cultures were exposed to 4-phenylbutylic acid (4PBA) or PP2 and then stimulated with TGF-*β*1. We found that E-cadherin, vimentin, fibronectin, and *α*-SMA expression was increased in nasal polyps compared to inferior turbinates. TGF-*β*1 increased the expression of EMT markers such as E-cadherin, fibronectin, vimentin, and *α*-SMA and ER stress markers (XBP-1s and GRP78), an effect that was blocked by PBA or PP2 treatment. 4-PBA and PP2 also blocked the effect of TGF-*β*1 on migration of A549 cells and suppressed TGF-*β*1-induced expression of EMT markers in PNECs and organ cultures of inferior turbinate. In conclusion, we demonstrated that 4PBA inhibits TGF-*β*1-induced EMT via the c-Src pathway in A549 cells, PNECs, and inferior turbinate organ cultures. These results suggest an important role for ER stress and a diverse role for TGF-*β*1 in upper airway chronic inflammatory disease such as CRS.

## 1. Introduction

Epithelial-mesenchymal transition (EMT) is a biological process that transforms a polarized epithelial cell into a mesenchymal cell phenotype. EMT is characterized by enhanced mobility and invasiveness and increased the production of extracellular matrix (ECM) components. EMT makes epithelial cells lose characteristics such as cell-to-cell adhesion and apical-basal polarity [1]. EMT contributes to the formation of tissues and organs during development and to wound healing by altering cell-ECM interactions, cytoskeletal organization, and cellular metabolism in a well-coordinated manner [2, 3]. However, dysregulated EMT can occur during chronic inflammation, leading to the disruption of epithelial integrity, disorganization of epithelial tissue, and production of malfunctioning mesenchymal cells and causing pathological remodeling and cancer progression [4–6]. Recently, there has been an emerging opinion that the remodeling and EMT observed in chronic rhinosinusitis (CRS) are the cause of disease recalcitrance [7, 8].

The endoplasmic reticulum (ER) is an organelle where secretory and membrane proteins are assembled into their secondary and tertiary structures. Several pathological conditions such as hypoxia, nutrient deprivation, and infection lead to accumulation of unfolded proteins in the ER (a process known as ER stress). Under ER stress, cells activate signaling cascades known as the unfolded protein response (UPR). The UPR comprises three major signaling pathways mediated by protein kinase-like ER kinase (PERK), activating transcription factor 6 (ATF6), and inositol-requiring enzyme 1 (IRE1) [9, 10]. Through these signaling pathways, UPR leads to attenuation of ER stress and restores ER function via general translational suppression, induction of ER chaperones, and upregulation of ER-associated degradation. XBP-1 and GRP78 are related to IRE1 activation and transcriptional activation of the UPR and are therefore recognized as reliable indicators of ER stress [11].

Tumor growth factor- (TGF-) *β*1 is a multifunctional peptide that regulates proliferation, differentiation, adhesion, migration, and other cellular functions and is involved in the pathogenesis of a variety of airway diseases related to remodeling, including CRS [12]. In a previous study, we confirmed that TGF-*β*1 causes EMT in the airway epithelium and nasal tissues [13]. Recent studies showed that ER stress also induces EMT in a variety of different cell types, such as alveolar epithelial cells and thyroid epithelial cells [14–16]. c-Src, a member of the Src family of tyrosine kinases, has been proposed as a possible target of UPR to induce EMT [17]. Based on these facts, we hypothesized that ER stress could be involved in TGF-*β*1-induced EMT and that c-Src could be involved in this process in cells and tissues of airway including the nose. Thus, in the present study, we investigated the role of ER stress and c-Src in TGF-*β*1-induced EMT.

## 2. Materials and Methods

### 2.1. Materials

Human recombinant TGF-*β*1 was obtained from R&D Systems (Minneapolis, MN) and dissolved in 0.1% bovine serum albumin (Millipore Inc., Billerica, MA). 4-PBA and PP2 (Sigma, St. Louis, MO, USA) were dissolved in DMSO (Sigma) and then diluted to the desired concentrations with complete medium. DMSO (<0.1%) was added to the medium.

### 2.2. Harvesting Nasal Polyps and Inferior Turbinates

Six patients with chronic rhinosinusitis with nasal polyps were recruited from the Department of Otorhinolaryngology, Korea University College of Medicine, Korea. Twelve inferior turbinate tissues were harvested during endoscopic sinus surgery for benign tumors. Half of them were used for control, and others were used for organ culture and cell culture. The patients that have any history of allergies, asthma, or aspirin sensitivity or received medications such as steroids, NSAIDs, antihistamines, or antibiotics within 4 weeks prior to tissue harvest were excluded. The Institutional Review Board of Korea University Guro Hospital approved the present study (KUGGR-12041-001), and informed consent was obtained from each patient.

### 2.3. Cell Culture

A549 cells, a human cell line derived from the respiratory epithelium, were purchased from the American Type Culture Collection (Manassas, VA). A549 cells were cultured in RPMI-1640 medium supplemented with 10% (*v/v*) heat-inactivated fetal bovine serum (Invitrogen, Carlsbad, CA), 1000 *μ*g/mL streptomycin (Invitrogen), and 1000 unit/mL penicillin.

For culture of primary nasal epithelial cells (PNECs), nasal tissues were washed with phosphate-buffered saline (PBS), digested in dispase (Stem Cell Technologies, Vancouver, Canada) for 4 hours, and then filtered through a mesh. PNECs were cultured in Bronchial Epithelial Cell Growth Medium (Lonza, Basel, Switzerland).

### 2.4. Inferior Turbinate Organ Culture

Inferior turbinates were dissected into three 3 mm pieces with scissors in sterile manner. Dissected tissues were washed three times with PBS and then placed onto a hydrated gelatin sponge (10 mm × 10 mm × 1 mm; Spongostan, Johnson & Johnson, San Angelo, TX) in 6-well plates. Each well was filled with 1.5 mL Dulbecco's Modified Eagle Medium (Invitrogen) including 2% fetal bovine serum (Invitrogen). Inferior turbinate tissues were stimulated with TGF-*β*1 (5 ng/mL) with or without 4PBA or PP2. Plates were incubated at 37°C in 5% CO_2_.

### 2.5. MTT (3-[4,5-Dimethylthiazol-2-yl]-2,5-diphenyl Tetrazolium Bromide) Assay

A549 cells or PNECs were placed onto 96-well plates at a density of 4 × 10^5^ cells/mL with various concentrations of 4PBA (0–20 mM) for 72 hours. Cells were incubated with MTT (Sigma) for 4 hours, and the reaction was stopped by the addition of acidified isopropanol. The results were measured by a fluorescence microplate reader (F2000; Hitachi Ltd., Tokyo, Japan) at 570 nm.

### 2.6. Immunofluorescence

Cells were fixed with 4% paraformaldehyde, permeabilized with 0.2% Triton X-100 in 1% bovine serum albumin for 10 minutes, blocked with 5% bovine serum albumin for 1 hour at room temperature, and incubated overnight at 4°C with monoclonal (vimentin and *α*-SMA) or polyclonal (E-cadherin and fibronectin) primary antibodies (Santa Cruz, CA). Cells were incubated with Alexa 488 anti-mouse IgG antibody or Alexa 555 anti-rabbit IgG secondary antibodies (Invitrogen, Carlsbad, CA) and counterstained with 4′,6-diamidino-2-phenylindole (Invitrogen, Carlsbad, CA). Images of immunostained cells were captured using a confocal microscope (LSM700; Zeiss, Oberkochen, Germany).

### 2.7. Reverse Transcription-Polymerase Chain Reaction (RT-PCR)

Total RNA was extracted using TRIzol reagent (Invitrogen), according to the recommendations of the manufacture. RT was carried out with 2 *μ*g RNA for each sample. MMLV reverse transcriptase (Invitrogen) was used according to the manufacturer's protocol. PCR was performed using the following primers: *GRP78* (sense sequence 5′- GTT CTT GCC GTT CAA GGT GG -3′ and antisense sequence 5′- TGG TAC AGT AAC AAC TGC ATG GG -3′, 180 bp), *XBP-1s* (sense sequence 5′- CCT GGT TGC TGA AGA GGA GG -3′ and antisense sequence 5′- CCA TGG GGA GAT GTT CTG GAG -3′, 138 bp), and *GAPDH* (sense sequence 5′- GTG GAT ATT GTT GCC ATC AAT GAC C -3′ and antisense sequence 5′- GCC CCA GCC TTC TTC ATG GTG GT -3′, 271 bp). Gels were visualized using a Molecular Imager ChemiDoc XRS+ (Bio-Rad, Hercules, CA).

### 2.8. Western Blot Assay

PRO-PREPTM protein extraction solution (iNtRON Biotechnology, Seongnam, Korea) was used to lyse cells or tissues. Proteins were separated by 10% sodium dodecyl sulfate polyacrylamide gel electrophoresis and transferred onto polyvinyl difluoride membranes (Millipore Inc., Billerica, MA). Membranes were blocked with 5% skim milk and incubated with the following antibodies: E-cadherin, vimentin, *α*-SMA, fibronectin, GRP78, XBP-1s, phosphorylated c-Src, and *β*-actin (Santa Cruz). The blots were detected after incubation with horseradish peroxidase-conjugated secondary antibodies by an enhanced chemiluminescence detection system (Pierce, Rockford, IL).

### 2.9. Cell Migration Scratch Assay

On 6-well tissue culture dishes, A549 cells were plated and cultured to confluence. Through the cells, a straight scratch was made with a pipette tip. Scratched cells were washed immediately with PBS, and culture medium was added. Cells were treated only with TGF-*β*1 (5 ng/mL) or in combination with 4PBA or PP2. After 48 hours, images were captured with a microscope (Olympus BX51; Olympus, Tokyo, Japan).

### 2.10. Transwell Migration Assay

Cells were placed onto the top chamber of a transwell chambers (Corning Life Sciences, MA). Chamber was filled with RPMI-1640 medium supplemented with 10% (*v/v*) heat-inactivated fetal bovine serum, 1000 *μ*g/mL streptomycin, and 1000 unit/mL penicillin (Invitrogen). TGF-*β*1 (5 ng/mL) alone or in combination with 4PBA or PP2 was added in the bottom chamber for 48 hours. Cells on the upper surface of the membrane were removed using a cotton swab, and cells on the lower surface were stained with Diff-Quik stain (Sysmex, Kobe, Japan). Images of stained cells from five selected fields of view were obtained using a microscope at 400x magnification.

### 2.11. Statistical Analysis

At least three independent experiments provided the results in this study. Unpaired two-way analysis of variance (ANOVA) test or one-way ANOVA followed by Tukey's test (GraphPad Prism, version 5, GraphPad Software, San Diego, CA) was used to confirm the Statistical significance of differences between control and experimental data. A 95% confidence level established significance. *P* values less than 0.05 were accepted as statistical significant.

## 3. Results

### 3.1. Reduced Expression of Epithelial Markers and Increased Expression of Mesenchymal EMT Markers in Nasal Polyp Tissues

**Figure 1:**
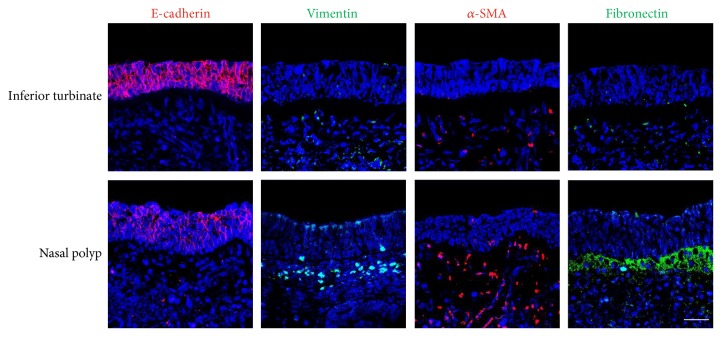
Expression of epithelial-mesenchymal markers E-cadherin, vimentin, *α*-smooth muscle actin, and fibronectin in nasal polyp and control (normal inferior turbinate) tissues determined by immunofluorescence. Scale bar = 50 *μ*m.

To investigate whether EMT occurs in CRS tissues in vivo, we examined the fluorescent immunocytochemical expression of the EMT markers including E-cadherin, vimentin, *α*-SMA, and fibronectin—in six nasal polyps and six normal inferior turbinates. E-cadherin expression was reduced in epithelial cells from nasal polyps compared to inferior turbinate epithelial tissues, as characterized by weak membrane staining. Staining for the mesenchymal markers vimentin, *α*-SMA, and fibronectin in nasal polyps was strongly positive in submucosal tissues and weaker on the apical epithelial side. However, in the inferior turbinate tissues, both epithelium and submucosa were negative for these mesenchymal markers (Figure 1).

### 3.2. 4-PBA Reduces TGF-*β*1-Induced Expression of ER Stress Markers in A549 Cells

**Figure 2:**
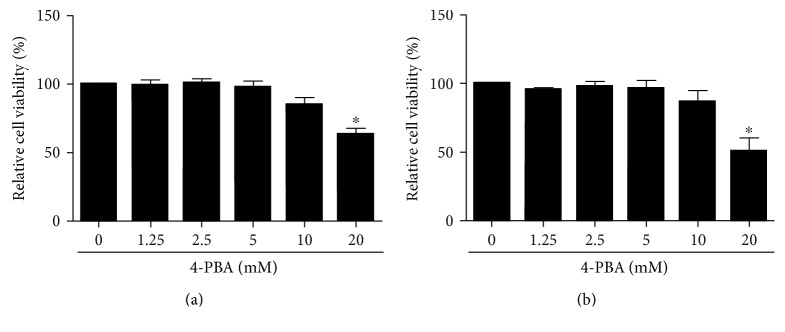
Cytotoxicity of 4-PBA determined by MTT assay in A549 cells (a) and in primary nasal epithelial cells (b). 4-PBA, 4-phenylbutyric acid. MTT, 3-[4,5-dimethylthiazol-2-yl]-2,5-diphenyl tetrazolium bromide. ^∗^*P* < 0.05 versus control.

**Figure 3:**
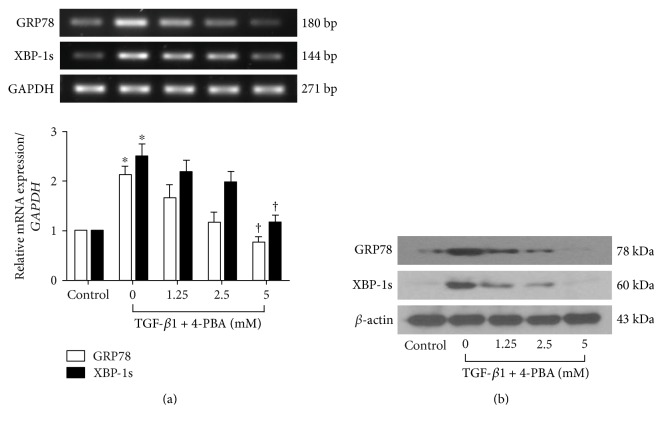
Effect of 4-PBA on GRP78 and XBP-1s mRNA and protein expression in TGF-*β*1-stimulated A549 cells determined by RT-PCR (a) and Western blotting (b) (representative of independent experiments). Values expressed as mean ± SEM of independent experiments. ^∗^*P* < 0.05 versus control. ^†^*P* < 0.05 versus TGF-*β*1 alone. 4-PBA, 4-phenylbutyric acid. GAPDH, glyceraldehyde-3-phosphate dehydrogenase.

To determine whether TGF-*β*1 causes ER stress in A549 cells, expression levels of the ER stress markers, GRP78 and XBP-1s, were measured using RT-PCR and Western blot. TGF-*β*1 induced the expression of GRP78 and XBP-1s mRNA after 24 hours, and protein expression was increased after 48 hours. Next, we investigated the effects of 4-PBA, a chemical chaperone, on TGF-*β*1-induced ER stress in A549 cells. Prior to treatment with 4-PBA, an MTT assay was performed on A549 cells and nasal epithelial cells to examine the effects of 4-PBA on cell survival. Cells were examined after treatment with 4-PBA concentrations ranging from 0 to 20 mM, and cell survival was not affected below 10 mM (Figure 2). After pretreatment with 4-PBA (1.24–5 mM) for 1 hour, cells were treated with TGF-*β*1 for 24 or 48 hours and the expression of GRP78 and XBP mRNA and protein were measured. Pretreatment with 4-PBA reduced the TGF-*β*1-induced expression of GRP78 and XBP-1s in a dose-dependent manner (Figure 3).

### 3.3. 4-PBA Inhibits TGF-*β*1-Induced EMT in A549 Cells

**Figure 4:**
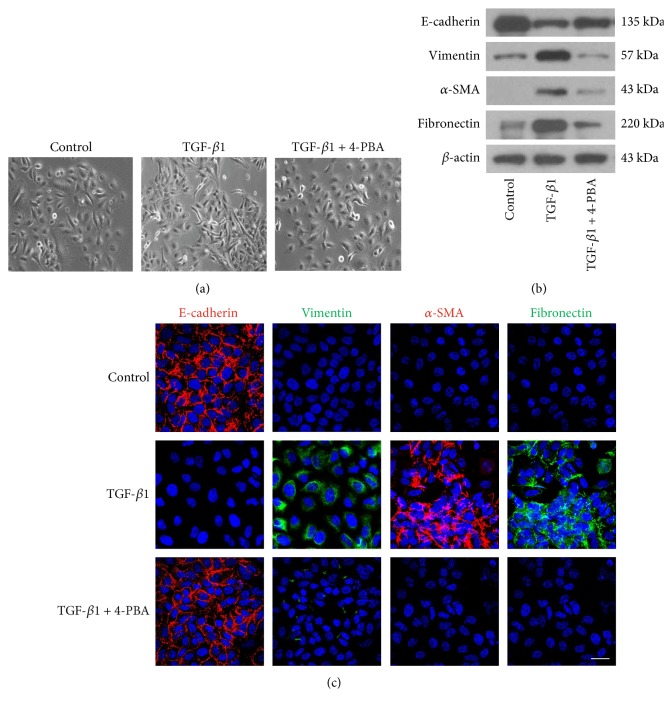
(a) Effect of 4-PBA on morphology of TGF-*β*1-stimulated A549 cells as observed under a phase-contrast microscope. Effects of 4-PBA on E-cadherin, vimentin, *α*-smooth muscle actin protein, and fibronectin expression in TGF-*β*1-stimulated A549 cells determined by Western blotting (b) and immunofluorescence (c). Representative of independent experiments. Scale bar = 50 *μ*m. 4-PBA, 4-phenylbutyric acid.

In a previous study, we showed that TGF-*β*1 induces EMT in the primary airway epithelial cells [13]. Cells were treated with 5 ng/mL TGF-*β*1 for 72 hours, and morphological changes of the cells were observed by phase-contrast microscopy. After 72 hours of TGF-*β*1 treatment, the normal cobblestone-like appearance of the epithelial cells was converted into a morphology of mesenchymal cells with an abnormal elongated appearance. Pretreatment of these TGF-*β*1-stimulated A549 cells with 4-PBA for 1 hour restored the morphology of normal epithelial cells (Figure 4(a)). We examined E-cadherin, vimentin, *α*-SMA, and fibronectin protein expression, as markers of EMT, using Western blot and fluorescent immunocytochemical staining (Figures 4(b) and 4(c)). TGF-*β*1 treatment for 72 hours decreased E-cadherin expression and increased vimentin, fibronectin, and *α*-SMA expression. Thus, 4-PBA reversed the effects of TGF-*β*1 on EMT in A549 cells.

### 3.4. TGF-*β*1-Induced ER Stress and EMT Are Mediated through c-Src Kinase Activation in A549 Cells

**Figure 5:**
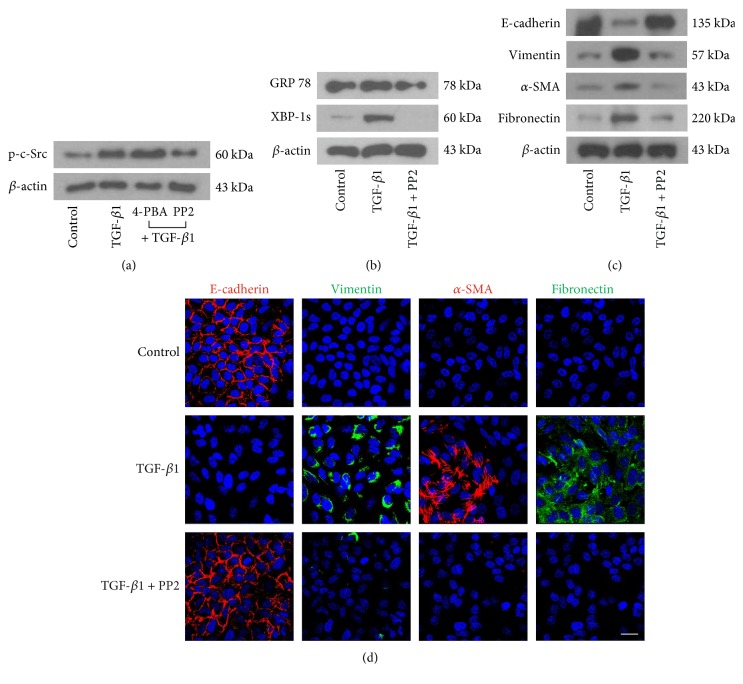
(a) Effect of 4PBA and PP2 on c-Src phosphorylation in TGF-*β*1-stimulated A549 cells determined by Western blotting (representative of independent experiments). (b) Effects of PP2 on GRP78 and XBP-1s protein expression in TGF-*β*1-stimulated A549 cells determined by Western blotting (representative of independent experiments). Effects of PP2 on E-cadherin, vimentin, *α*-smooth muscle actin protein, and fibronectin expression in TGF-*β*1-stimulated A549 cells determined by Western blotting (c) and immunofluorescence (d). Representative of independent experiments. 4-PBA, 4-phenylbutyric acid.

To evaluate the contribution of c-Src kinase to TGF-*β*1-induced ER stress and EMT, we used the c-Src kinase inhibitor, PP2 (2 *μ*M). We first confirmed that phosphorylation of c-Src kinase is stimulated by TGF-*β*1 (5 *μ*M) in A549 cells and then determined whether it was blocked by PP2 treatment. Phosphorylation of c-Src kinase was increased in TGF-*β*1-treated A549 cells after 48 hours, as shown by Western blotting, and this was reduced by PP2 (2 *μ*M) but not by 4-PBA (5 mM) (Figure 5(a)). PP2 treatment inhibited the TGF-*β*1-induced changes in ER stress marker proteins (GRP78 and XBP-1) after 48 hours and in EMT marker proteins (E-cadherin, vimentin, *α*-SMA, and fibronectin) after 72 hours (Figures 5(b) and 5(c)). Localization of the EMT markers was observed using fluorescent immunocytochemical staining, and these results mirrored those from the Western blotting (Figure 5(d)). These data indicate that c-Src is involved in TGF-*β*1-induced ER stress and EMT in A549 cells.

### 3.5. 4-PBA and PP2 Inhibit Migration of TGF-*β*1-Induced A549 Cells

**Figure 6:**
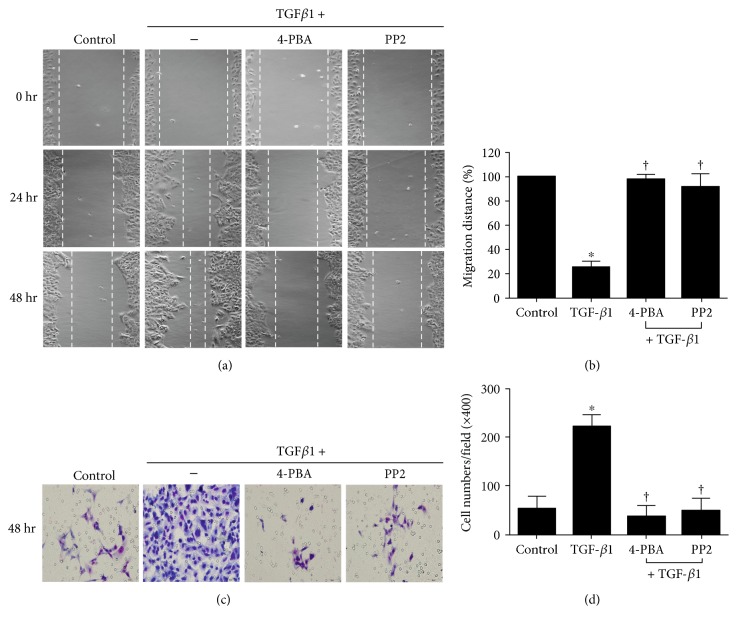
Effect of 4-PBA and PP2 on migratory ability of TGF-*β*1-stimulated A549 cells measured using cell migration (a, b) and transwell invasion (c, d) assays. Values expressed as mean ± SEM of independent experiments. ^∗^*P* < 0.05 versus control. ^†^*P* < 0.05 versus TGF-*β*1 alone. Scale bar = 50 *μ*m.

As one of the important functional characteristics of mesenchymal cells is increased migratory ability, cell migration assay was done to measure changes in the migratory capacity of A549 cells. We made a straight scratch in the middle of adherent cells on the plate using a pipette tip. And then, we stimulated the cells with TGF-*β*1, with or without 4-PBA or PP2, and determined the distance from the initial boundary to the moved cells. After 48 h, cells had moved meaningfully further from the boundary of the initial scratch in TGF-*β*1-treated cells, compared to controls. This cell migration was inhibited following pretreatment with either 4-PBA or PP2 (Figures 6(a) and 6(b)). We reevaluated the inhibitory effect of 4-PBA and PP2 on the increased migratory ability of TGF-*β*1-induced A549 cells by performing a transwell invasion assay. Cells were treated with TGF-*β*1 with or without 4-PBA or PP2 for 48 h, and the number of cells that had moved through the filter and adhered to the underside of the well was counted. These results confirm that pretreatment with 4-PBA and PP2 blocks the enhanced cell invasion seen in TGF-*β*1-treated cells (Figure 6(b)). In summary, the increased migratory ability induced by TGF-*β*1 is blocked by a chemical chaperone of ER stress and inhibition of c-Src phosphorylation.

### 3.6. 4-PBA and PP2 Inhibit TGF-*β*1-Induced EMT in PNACs and Nasal Inferior Turbinate Organ Cultures

**Figure 7:**
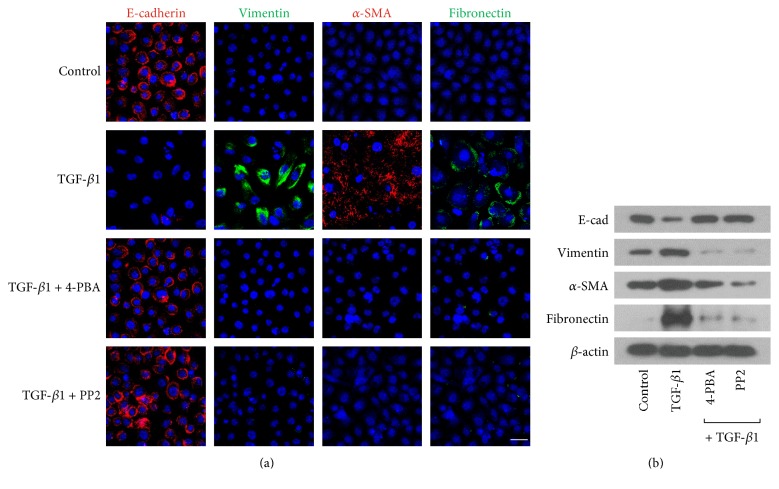
(a) Effect of 4-PBA and PP2 on E-cadherin, vimentin, *α*-smooth muscle actin protein, and fibronectin expression in TGF-*β*1-stimulated primary nasal epithelial cells determined by immunofluorescence. (b) Effects of 4-PBA and PP2 on E-cadherin, vimentin, *α*-smooth muscle actin protein, and fibronectin expression in TGF-*β*1-stimulated inferior turbinate tissues determined by Western blotting. Representative of independent experiments. Scale bar = 50 *μ*m. 4-PBA, 4-phenylbutyric acid.

To determine whether the blockage of TGF-*β*1-induced EMT by PBA and PP2 in A549 cells is also seen in nasal tissue, we repeated our experiments in PNECs and inferior turbinate organ cultures. To access whether TGF-*β*1 causes EMT in PNECs, cells were treated with 5 ng/mL TGF-*β*1 for 72 hours and then we observed expression of fibronectin, *α*-SMA, vimentin, and E-cadherin protein using a fluorescence microscope. The cells showed increased vimentin, *α*-SMA, and fibronectin expression and decreased E-cadherin expression. 4-PBA or PP2 pretreatment for an hour blocked the effects of TGF-*β*1 on EMT in PNECs (Figure 7(a)). In nasal inferior turbinate organ cultures, tissues were exposed to TGF-*β*1 for 72 hours, with or without 4-PBA or PP2, and expression levels of *α*-SMA, fibronectin, vimentin, and E-cadherin protein were assayed using Western blot. Expression levels of *α*-SMA, vimentin, fibronectin were increased, and E-cadherin expression was decreased in TGF-*β*1-treated inferior turbinate organ cultures, compared to controls. However, pretreatment with PBA or PP2 inhibited the effect of TGF-*β*1 on expression of EMT markers. These results show that 4-PBA or PP2 pretreatment ameliorate EMT induced by TGF-*β*1 in cells and tissues of the nose (Figure 7(b)).

## 4. Discussion

In the present study, we first confirmed that features of EMT are increased in nasal polyp tissues and then showed that a chemical chaperone of ER stress, 4PBA, inhibits TGF-*β*1-induced EMT in A549 cells, PNECs, and inferior turbinate organ cultures. TGF-*β*1 increased mRNA and protein expression levels of ER stress markers (XBP-1s and GRP78) and also altered expression levels of EMT markers, namely, E-cadherin, vimentin, fibronectin, and *α*-SMA. Pretreatment with 4PBA reversed the effect of TGF-*β*1 on EMT in A549 cells, while pretreatment with PP2 reversed the effect on both ER stress and EMT. However, 4PBA treatment did not show inhibitory effects on c-Src phosphorylation in TGF-*β*1-induced A549 cells. 4PBA and PP2 also reversed the stimulatory effect of TGF-*β*1 on the migratory and invasive ability of the cells, which was a characteristic of mesenchymal cells in both a cell migration and transwell invasion assay. In experiments using PNECs and inferior turbinate tissues, 4PBA and PP2 suppressed the changes in EMT marker expression levels that were induced by TGF-*β*1.

Inflammation leads to a varied degree of tissue injury, depending on the disease and its severity. This means that remodeling occurs in all inflammatory disease, as remodeling is an essential process in the healing and repair of injured tissue. Remodeling that occurs in response to a minor inflammatory condition usually leads to a normal reconstructive process. On the contrary, dysregulated remodeling, such as that caused by severe or chronic long-lasting inflammation, can cause pathological reconstruction and formation of pathological tissue [18]. Pathological remodeling of the lower airways has received considerable attention as it is one of the major features of asthma and chronic obstructive pulmonary disease. As a result, much progress has been made towards understanding these diseases [19]. The role of remodeling in the upper airway chronic inflammatory disease, such as CRS, has received less attention. This could be due in part to the fact that remodeling of the upper airway does not result in the same fatal airflow limitations that occur in patients with asthma and chronic obstructive pulmonary disease. Recently, however, there has been increased concern regarding the role of remodeling in the upper airway diseases, including CRS, for two main reasons. The first is that the upper airway remodeling can lead to irreversible structural changes, which could explain the recalcitrance of disease [20, 21]. The other is that remodeling begins early in the development of disease and therefore plays an important role in the disease progression. Although remodeling in CRS has been considered a secondary process that results from persistent inflammation over a long period of time, a recent study showed that remodeling actually occurs in parallel with inflammation [22]. As a result, remodeling is now thought to be one of the main reasons for disease recalcitrance, and EMT is receiving great attention as a convergence point between inflammation and pathological remodeling in many progressive fibrotic diseases. Accumulating evidence has confirmed that ongoing EMT leads to loss of epithelial barrier function, which would also contribute to disease progression [12].

Endoplasmic reticulum (ER) stress refers to the state where protein folding in the ER is disrupted by alterations in homeostasis within the ER lumen. UPR is the main mechanism by which cells adapt to ER stress and maintain homeostasis. In recent years, ER stress has been implicated in fibrotic remodeling during chronic inflammatory disease [23, 24]. Furthermore, studies suggest that ER stress induces EMT in a number of biological systems [25]. Based on these facts, we sought to evaluate the effect of TGF-*β*1, a known strong inducer of EMT, on ER stress. In present study, we showed that TGF-*β*1 simultaneously affected the expression levels of ER stress markers and EMT markers in A549 cells and that these effects were reversed by the chemical chaperon of ER stress, 4PBA. To our knowledge, this is the first study to demonstrate a role for TGF-*β*1 as an inducer of both ER stress and EMT.

The Src protein nonreceptor tyrosine kinase family is a group of cytoplasmic tyrosine kinases capable of communicating with a large number of different receptors and thus regulates many cellular events [26]. c-Src is a member of the Src tyrosine kinase family, and it plays a critical role as a switch in mediating signal transduction via interactions with multiple proteins and protein complexes. Abnormal activation of c-Src is involved in apoptosis, proliferation, cell adhesion, cell migration, and invasion, and all of which are related to tumor progression [27]. Dysfunction of E-cadherin caused by c-Src could also contribute to our understanding of EMT. In fact, increased Src activity promotes EMT, while c-Src inhibition suppresses this process [28]. In the present study, we showed that c-Src inhibition by PP2 reversed the effect of TGF-*β*1 on EMT in airway epithelial cells. Moreover, PP2 also inhibited the expression of ER stress markers, XBP-1 and GRP78. We can therefore assume that c-Src is involved in TGF-*β*1, UPR, and the EMT axis at a higher level than UPR.

## 5. Conclusions

We demonstrated that 4PBA inhibits TGF-*β*1-induced EMT via the c-Src pathway in A549 cells, PNECs, and inferior turbinate organ cultures. These results suggest an important role for ER stress and a diverse role for TGF-*β*1 in the upper airway chronic inflammatory diseases such as CRS. In the near future, it will also be necessary to demonstrate additional level of evidences done with diseased tissue instead of TGF-*β*1-induced tissue. We hope these approach elucidate the role of ER stress modulation in the treatment of recalcitrant CRS related to tissue remodeling.
